# TRANSPARENT TESTA GLABRA 1-Dependent Regulation of Flavonoid Biosynthesis

**DOI:** 10.3390/plants6040065

**Published:** 2017-12-20

**Authors:** Bipei Zhang, Andrea Schrader

**Affiliations:** Botanical Institute, University of Cologne, Zuelpicher Str 47B, 50674 Cologne, Germany; bzhang1@smail.uni-koeln.de

**Keywords:** *Arabidopsis thaliana*, TRANSPARENT TESTA GLABRA 1, TTG1, WD40, flavonoid biosynthesis, MBW complex, MYB, bHLH

## Abstract

The flavonoid composition of various tissues throughout plant development is of biological relevance and particular interest for breeding. *Arabidopsis thaliana* TRANSPARENT TESTA GLABRA 1 (AtTTG1) is an essential regulator of late structural genes in flavonoid biosynthesis. Here, we provide a review of the regulation of the pathway’s core enzymes through AtTTG1-containing R2R3-MYELOBLASTOSIS-basic HELIX-LOOP-HELIX-WD40 repeat (MBW(AtTTG1)) complexes embedded in an evolutionary context. We present a comprehensive collection of *A. thaliana* *ttg1* mutants and *AtTTG1* orthologs. A plethora of MBW(AtTTG1) mechanisms in regulating the five major *TTG1*-dependent traits is highlighted.

## 1. Motivation

Flavonoids are secondary plant metabolites with a broad spectrum of functions for plants, including UV protection, pollination and feeding attraction, rhizosphere signaling and pathogen defense [1,2,3,4,5]. Early on, health-promoting effects of flavonoids were under investigation, and a mixture of flavonoids from citrus was initially named “vitamin P” by Szent-Györgyi in 1936 [6].

Generations of researchers have been attracted by flavonoids, their function and the regulation of their biosynthesis pathways. Potential health-promoting benefits for human nutrition, as well as medical topics like binding and inhibition of proteins, absorption, conversion and localization within the human body were in the focus of more than 70% of flavonoid reviews. Apart from these, two major types of topics dominated other reviews since 1964: (1) the substances themselves (e.g., lists of substances, identification of individual substances, chemical properties, synthesis and conversion, analytical methodology, composition in specific species, tissues and developmental stages) and (2) their function (e.g., functions tested in vitro, like radical scavenging, functions in plants like UV protection and functions related to the plant′s environment like exudate signaling within the rhizosphere, pathogen defense, pollination or feeding attraction). 

Different types of flavonoids are known to have different functions (e.g., [3,7,8,9,10]). Therefore, when it comes to breeding efforts, understanding the varying flavonoid composition of specific plant organs throughout development is of utmost relevance. So far, only a few reviews have centered on specific aspects of the flavonoid biosynthesis pathway’s regulation through plant development. 

### Where We Are and Where to Go?

Screening for plants with deviating coloration of plant organs facilitated the identification of the core pathway’s enzymes and essential regulators. These mutant sets, combined with sequencing technology, have already answered the “Who is who?” and “Who does what?” questions for the flavonoid biosynthesis core pathway (e.g., summarized in [11,12,13,14,15,16]).

We can now specify and ask “Who does what, where, when and why?” It is important to note that the functional spatiotemporal focus moves the gene function of regulators and their own regulation to center stage. AtTTG1 (*Arabidopsis thaliana* (*A. thaliana*) TRANSPARENT TESTA GLABRA 1) is one of these regulators. With the *AtTTG1*-dependent gene regulatory network, it acts as a regulatory hub, differentially modulating spatiotemporal flavonoid composition of plant tissues throughout development and is an excellent example for such concerted regulatory mechanisms.

Moving away from solved “yes-no” scenarios of the past, in which the identified defective factor of a mutant would block the whole downstream pathway (e.g., of *A. thaliana tt* (*transparent testa*) mutants), it will now be important to complement these findings with more subtle, but relevant modifications of the pathway, e.g., accessible through natural variation studies. At the same time, gene function and regulation should be linked not only with individual substances, but also with flavonoid composition of specific plant organs in various species, which is of interest for plant breeding purposes. 

Flavonoids have been at the center of research for an impressive period of time by now. In the post-genomic era, novel questions can be asked, and the wealth of knowledge that stems from research in the well-established model species *A. thaliana* can be extended to other species. This requires and allows for an embedding of previous findings in an evolutionary context to drive future research projects and to link different areas of research.

Towards this end, this review provides a small contribution with a focus on *AtTTG1*, the AtTTG1-dependent regulation of the flavonoid biosynthesis and an embedding into the evolutionary context. Moreover, this review provides an overview of comprehensive collections of *A. thaliana ttg1* mutants and *AtTTG1* orthologs.

## 2. Introduction

### 2.1. Flavonoid Biosynthesis Pathway 

At the end of the last century, *A. thaliana*, an annual Brassicaceae with a small genome, emerged as the model organism for molecular geneticists. Its genome was the first sequenced genome of a flowering plant and was released in the year 2000 [17]. Extensive detailed knowledge about the flavonoid biosynthesis pathway and its regulator stems from this workhorse of molecular genetics. Studies on this pathway in *A. thaliana* benefit from the fact that the core enzymes are only encoded by a single gene except for six copies for *FLAVONOL SYNTHASE* (*FLS*), out of which two were shown to be expressed [18,19]. 

**Figure 1:**
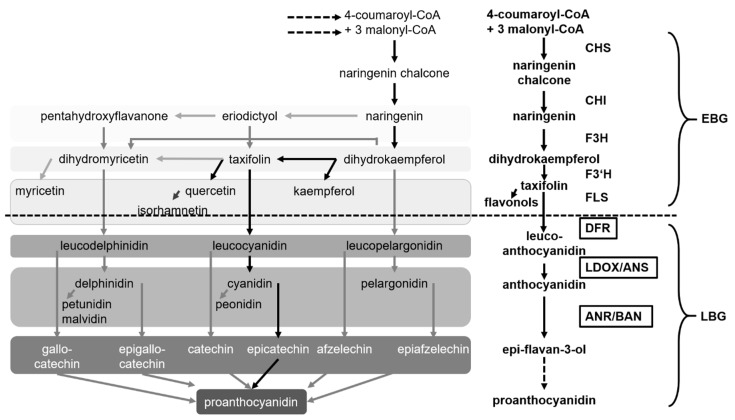
Flavonoid biosynthesis pathway of plants modified from [22] with an emphasis on *A. thaliana* seeds. Black arrows highlight the core pathway and substances in *A. thaliana* discussed in this review. The respective enzymatic steps from the different levels of the pathway are extracted on the right. Examples for different products of OMTs (O-METHYLTRANSFERASEs) are given (isorhamnetin, peonidin, petunidin, malvidin). Note that not all steps occur in *A. thaliana* and other substances can also occur in *A. thaliana*: e.g., pelargonidin. See [22] for details. The dashed arrow indicates that several proteins and enzymes are required for proanthocyanidin synthesis, deposition and/or further conversion to condensed tannins including transport processes (for a review on flavonoid transport and a few recent findings, see [24,25]). R2R3-MYELOBLASTOSIS-basic HELIX-LOOP-HELIX-WD40 repeat (MBW(AtTTG1)) complexes predominantly regulate late biosynthetic genes encoding for the core pathway enzymes. Dashed line: the assumed “border” between early and late biosynthetic genes and products in *A. thaliana* [20,26,27]. Boxed enzymes are under differential developmental regulation of MBW(AtTTG1) complexes. See Section 6 for details. Reduced levels of isorhamnetin in seeds of *ttg1-1*, but not *transparent testa* (*tt*)*2* and *tt8* mutants, indicate that the border might need some adjustment or that additional AtTTG1-dependent regulation occurs [22]. CHS: CHALCONE SYNTHASE, CHI: CHALCONE ISOMERASE, F3H: FLAVANONE 3-HYDROXYLASE, F3′H: FLAVONOID 3′ HYDROXYLASE, FLS: FLAVONOL SYNTHASE, DFR: DIHYDROFLAVONOL 4-REDUCTASE, LDOX: LEUCOANTHOCYANIDIN DIOXYGENASE, ANS: ANTHOCYANIDIN SYNTHASE, ANR: ANTHOCYANIDIN REDUCTASE, BAN: BANYLUS, EBG: early biosynthetic genes, LBG: late biosynthetic genes.

The flavonoid biosynthesis pathway in *A. thaliana* comprises core substances from several groups: e.g., chalcones, flavanones, flavonols, anthocyanidins, flavan-3-ols and proanthocyanidins. There are a few thousand derivatives of these substances (for more details, see Figure 1 and, e.g., [12,20,21]; for a fingerprint of the flavonoid/aglycon composition of *A. thaliana* seeds and seedlings, see [22] and its supplement).

Flavonoids are widely distributed within the plant kingdom and can be found in bryophytes, vascular plants and spermatophytes. It is discussed that flavonoids initially evolved as regulators or messengers and then as UV protectants [23]. The early steps of the pathway are shared by mosses and, e.g., angiosperms and therefore thought to be of ancient origin. As both parts of the pathway have evolved separately, but have evolved for a long time, it might not be surprising to find both early and late biosynthesis of enzymes: specific and overlapping sets of regulators. AtTTG1-dependent regulation is predominantly specific for the late biosynthesis of enzymes. 

### 2.2. Trait Complexity and the Evolution of Transcription Factor Families

With increased trait complexity in plant organisms, transcription factor gene families have expanded [28]. For bHLH (basic HELIX-LOOP-HELIX), as well as for many other transcription factors, a strong expansion accompanied the period of land invasion [29]. Transcription-associated proteins (TAPs) can be separated into transcription factors (like R2R3-MYB(MYELOBLASTOSIS (homolog)) or bHLH), binding cis-regulatory elements in a sequence-specific manner and transcription regulators (like TTG1) that are of relevance within gene regulatory networks, but do not necessarily bind DNA themselves [28]. The combinatorial control in transcription factor complexes with components from different transcription factor classes allowed the integration of different developmental and environmental signals for further trait diversification [30]. In agreement with this, transcription factor family expansion correlated with morphological complexity in land plant evolution. This was not the case for transcriptional regulators [28].

Within the *AtTTG1*-dependent gene regulatory network, the highest trait specificity is found for the R2R3-MYB factors (see the next section), indicating that these might have evolved the most towards specific adaptive traits. A reduced complexity is seen for bHLH factors with partially overlapping function. Differential complex composition of MBW(AtTTG1) complexes can explain the differential AtTTG1-dependent regulation of the flavonoid biosynthesis pathway. In particular, differing DNA binding motif preferences of the respective R2R3-MYB components have been reported [31,32,33].

## 3. *AtTTG1*-Dependent Gene Regulatory Network

**Figure 2:**
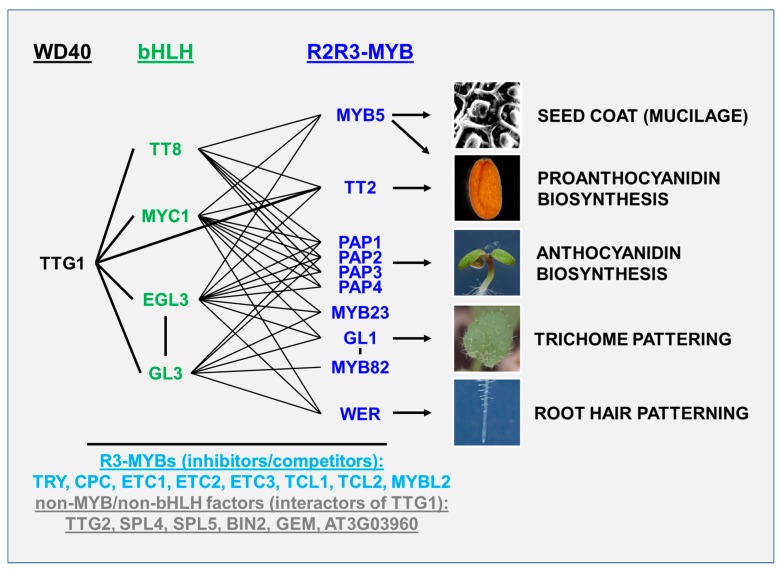
*AtTTG1*-dependent gene regulatory network. Proteins encoded by the *AtTTG1*-dependent gene regulatory network and possible differential MBW(AtTTG1) complex formation, regulating the five major *AtTTG1*-dependent traits. Shown are all bHLH and R2R3-MYB proteins that can form a ternary complex with AtTTG1 and regulate one of the five major *AtTTG1*-dependent traits indicated on the right. Lines represent interactions as documented in the BioGrid database (www.thebiogrid.org) [39]. Inhibitors/competitors compete with respective R2R3-MYBs for binding to bHLH proteins. We also extracted from the BioGrid database non-MYB and non-bHLH factors that interact with TTG1 and added the recently found interactors SPL4 and SPL5. Interactions are documented in a variety of papers [40,41,42,43,44,45,46,47,48,49,50,51,52,53]. For the inhibitors, see e.g., [54,55,56]. The seed coat picture was acquired by Hans-Peter Bollhagen. Photography of the seedling was done by Siegfried Werth. TTG1: TRANSPARENT TESTA GLABRA 1, GL3: GLABRA3, EGL3: ENHANCER OF GLABRA3, MYC: MYELOCYTOMATOSIS (homolog), TT: TRANSPARENT TESTA, WER: WERWOLF, MYB: MYELOBLASTOSIS (homolog), PAP: PRODUCTION OF ANTHOCYANIN PIGMENT, TRY: TRIPTYCHON, CPC: CAPRICE, ETC: ENHANCER OF TRY AND CPC1, TCL: TRICHOMELESS, MYBL: MYB-like, TTG2: TRANSPARENT TESTA GLABRA 1, SPL: SQUAMOSA PROMOTER BINDING PROTEIN-LIKE, BIN2: BRASSINOSTEROID-INSENSITIVE 2, GEM: GL2-EXPRESSION MODULATOR. PAP3 and PAP4 are also known as MYB113 and MYB114, respectively [57].

*AtTTG1* is the head of an evolutionarily-conserved gene regulatory network, regulating five *AtTTG1*-dependent traits with adaptive value for the plant: (1) trichome and (2) root hair patterning, (3) accumulation of anthocyanidin (and its derivatives) (in seedlings), (4) seed coat pigmentation and (5) seed coat differentiation including mucilage production (Figure 2). Differential MBW(AtTTG1) complex composition underlies the regulation of the five major traits [15,20,34,35,36,37,38].

MBW(AtTTG1) complexes consist of three classes of proteins: R2R3-MYB, bHLH and the WD40 repeat protein TTG1. Complexes of higher order are possible due to the homo- and heterodimerization of bHLH proteins [40,42,58]. The highest specificity of the trait regulation by MBW(AtTTG1) complexes exists on the level of the R2R3-MYB factors, while the bHLH factors act redundantly towards the different traits, and AtTTG1 is indispensable for all five traits. While we focus on the flavonoid biosynthesis in this review, we would like to point to previous reviews covering the role of AtTTG1 in trichome and root hair patterning (e.g., [59,60]). AtTTG1 has also been positioned in the seed coat mucilage pathway [61].

## 4. The Components of MBW(AtTTG1) Complexes

### 4.1. R2R3-MYBs

MYB proteins are found in all eukaryotic organisms [62,63]; first identified in the v-*myb* oncogene of the avian myeloblastoma virus, later in the human proto-oncogene c-*myb* and other related factors. MYB proteins in general contain up to three imperfect repeats: R1, R2 and R3 with R2 and R3 representing the minimum DNA-binding domain and containing cooperative recognition helices [62,64,65,66,67]. Few plant 4R-MYB proteins are reported (e.g., in soy bean) [68,69,70]. R2R3-MYB proteins are the most abundant plant-specific MYB proteins [63,65,66,71]. A consensus motif is present in all R2R3-MYB domains from *A. thaliana* [72]. The first cloned plant MYB gene was *Colorless1* (*C1*) from maize (*Zea mays*) that regulates anthocyanin accumulation [73]. *c1* was the gene disrupted in McClintock’s experiments (e.g., [74]) underlying her discovery of transposable elements. Several reviews have focused on plant MYB transcription factors in general, specific MYB pathways’ regulation or species (see e.g., [32,69,70,75,76,77,78,79,80,81])

### 4.2. bHLHs

Plant bHLH proteins of subgroup IIIf and MYB proteins containing the bHLH interaction motif [DE]Lx_2_[RK]x_3_Lx_6_Lx_3_R directly interact [46]. Subgroup IIIf bHLH proteins are already present in mosses [29]. Similar to R2R3-MYB proteins, the first cloned plant bHLH proteins originated from maize. In 1989, the *R* (*Red 1*) and *B* (*Booster 1*) genes were cloned [82,83].

The bHLH motif was first discovered and described in murine muscle development transcription factors and found to mediate dimerization and DNA binding [84]. It consists of a basic region at the N-terminus that binds specific DNA motives and an HLH region that mainly forms homo- and hetero-dimers with bHLH proteins [85]. Several reviews have focused on plant bHLH transcription factors in general and in specific pathways (see, e.g., [81,86,87]).

### 4.3. WDRs—TTG1

WD40 repeat (WDR) proteins are conserved in eukaryotes [88]. They have evolved in plants in various protein families with diverse functions: e.g., signal transduction, cytoskeletal dynamics, chromatin modification or transcriptional regulation [88]. This is in part due to diversification of regulators and targets up- and downstream the WDR proteins that act as interaction platform, constituents for protein complexes and sites of transient protein contacts [88]. WDR proteins are characterized by different numbers of WD40 repeats (usually 4–10 in plants) [88]. WDR proteins share a stretch of about 40 amino acids that usually end with Trp-Asp (WD) in each WD40 repeat [89]. Four and more WD40 repeats in one protein can form so-called β-propellers, a cylindrical formed series of four-stranded antiparallel beta sheets [88,90]. In the mammalian G-protein subunit Gβ, it is shown that the first and last WD 40 repeat contribute to the same beta-blade [90]. There are 237 WDR proteins with more than four repeats in *A. thaliana* [88], including TTG1, CONSTITUTIVELY PHOTOMORPHOGENIC 1 (COP1) and the four SUPPRESSOR OF PHYA-105 (SPA) proteins [34,91,92,93].

*TTG1* sequences were known to be present in angiosperms, but not in gymnosperms and older plant lines [31]. This requires an update, as recently, an ortholog has been identified from Norway spruce (*Picea abies*) [94]. For an overview of (putative) *AtTTG1* orthologs, see Section 9 of this review.

## 5. *AtTTG1* Mutants

*A. thaliana* mutants of At*TTG1* were fundamental in elucidating its gene and proteins functions. One of the often used and stronger mutants is *ttg1-1*, which carries a mutation leading to a premature stop codon and thereby to a truncation of the AtTTG1 protein at its far C-terminus. A closer view of the *AtTTG1* gene and protein structure will help to understand the relevance of the C-terminus for AtTTG1.

In *AtTTG1* loss-of-function mutants, leaves are glabrous as no trichomes are formed, ectopic root hairs are additionally produced, seeds are yellow instead of brownish, seedlings do not accumulate anthocyanidin and the columella, as well as the mucilage of seed coat epidermal cells is missing. 

Both flavonoid-related *AtTTG1*-dependent traits, anthocyanidin accumulation and seed coat pigmentation melt down to a differential regulation of the flavonoid biosynthesis pathway. The TTG1 syndrome of *ttg1* mutants varies in dependence of the position and type of mutation. Table 1 lists 22 *A. thaliana ttg1* mutants and the observed flavonoid-related pigmentation phenotype of seeds.

**Figure 3:**
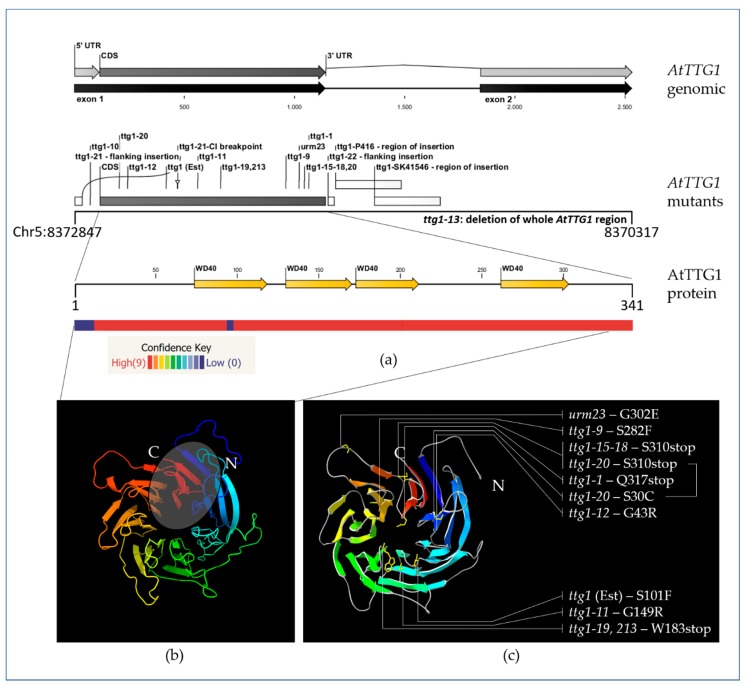
(**a**) *AtTTG1* gene structure, mutants and protein domains. The *AtTTG1* gene structure was retrieved from TAIR [105]. Exons, introns, 5′ and 3′UTR, mutants and protein domains were visualized using CLC DNA workbench 5.3.1 (CLC bio, Aarhus, Denmark). Please note that the intron is not part of the 3′UTR, which comprises the last nucleotide of exon 1 and the full exon 2. When searching databases for domains and repeats of AtTTG1, the resulting number, positioning and confidence for the detected WD40 repeats are diverse (e.g., SMART, PROSITE) [106,107,108,109]. Shown here is the annotation provided by UniProt. The colored bar below the protein sequence indicates the confidence of the Phyr2 model shown in (**b**) along the protein [110]. (**b**,**c**) AtTTG1 tertiary structure. The AtTTG1 protein tertiary structure was modeled using (**b**) Phyre2 or (**c**) loaded into SwissProt PdbViewer v.4.1.0 for marking the amino acids that are mutated or changed to a stop codon in the mutants [110,111]. Both (**b**,**c**) underline a part of the β-sheet structure, in particular the relevance of the C-terminus of AtTTG1 for the tertiary structure (highlighted by an ellipse in (**b**)). For the list of mutants and respective references, please refer to Table 1. CDS: coding DNA sequence, UTR: untranslated region.

The position of the mutations within these mutants indicates that proper AtTTG1 function requires an intact C-terminus. In Gβ, the first and last WD40 repeat contribute to the same β-blade; a similar scenario might occur in AtTTG1. Together with the modeled 3D-structure of AtTTG1, this points to a high relevance of the C-terminus for the protein’s proper folding and domain structure (see Figure 3). 

Interestingly, according to the reported insertion loci (Table 1; [105]), two of the activation tagging T-DNA insertions were shown to be located in intron 1. This might change the 3′UTR due to differential splicing and possible loss of the 3′UTR portion originating from exon 2 in the transcript (exon 1 includes the stop codon and the first nucleotide of the 3′UTR; exon 2 comprises the remaining 3′UTR; see Figure 3). Moreover, similar as recently reported for *GLABRA3* (*GL3*) [112], the intron might have a regulatory function, which could be impaired by the T-DNA insertion (size of inserted T-DNA > 6 kb). A T-DNA insertion in the *AtTTG1* intron might change the splicing, the 3’UTR and its function, transcript stability and movement properties. Furthermore, an effect of the activation tagging insert including 35S promoters and a possible additional transcript is possible.

Helen A. Stafford described the differential accumulation of proanthocyanidin between plants with different developmental strategies. While abundant in gymnosperms and some ferns, proanthocyanidins are still found as defense compounds in leaves of long-lived woody plants, and they are rare in short-lived, herbaceous plants in which they are found in the seed coat of some of these [23]. In *A. thaliana*, seeds off *ttg1* null mutants appear yellow due to the missing pigments [15]. They have a transparent testa. These pigments require the activity of DIHYDROFLAVONOL 4-REDUCTASE (DFR) and other downstream enzymatic steps [14]. These include LEUCOANTHOCYANIDIN DIOXYGENASE (LDOX)/ANTHOCYANIDIN SYNTHASE (ANS), ANTHOCYANIDIN REDUCTASE (ANR)/BANYLUS (BAN) and, in other species, also LEUCOANTHOCYANIDIN REDUCTASE (LAR) activity [20].

## 6. Target Enzymes of MBW(AtTTG1) Complexes in the Core Flavonoid Biosynthesis Pathway

**Figure 4:**
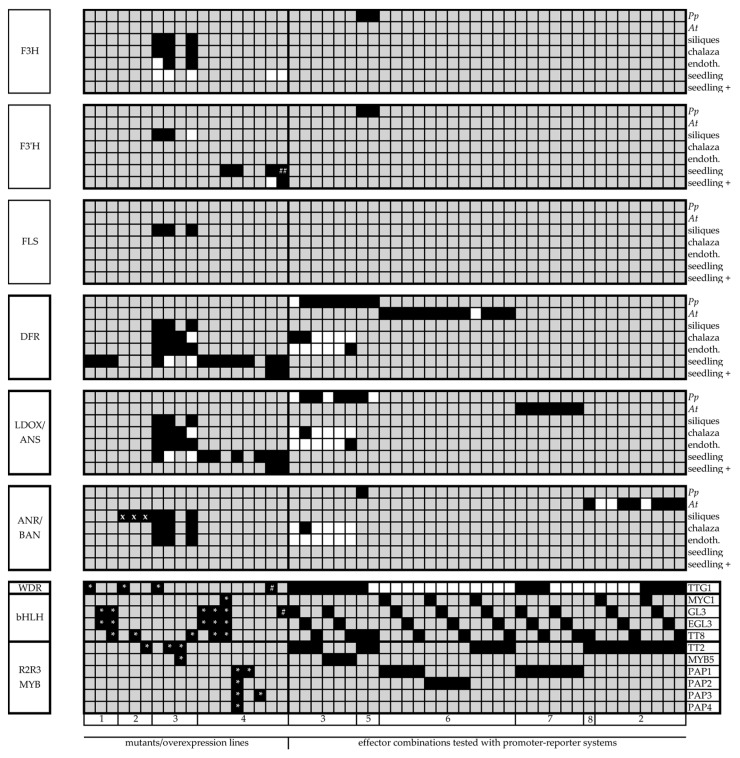
Tissue-specific MBW(AtTTG1) regulation of the flavonoid biosynthesis’ LBGs. Collection of exemplifying studies analyzing the flavonoids core enzyme’s regulation through MBW(AtTTG1) components. These comprise tissue-specific, mutant and promoter-reporter experimental approaches. Black boxes next to the MBW factors indicate the combinations of MBW factors, used mutants or overexpression lines in the respective experiment summarized above for the different LBG promoters. Black boxes next to the enzymes depend on the type of experiment. The respective enzyme’s promoter was either (1) upregulated by combinations of MBW factors or in MBW overexpression lines; (2) downregulated in the respective mutant; (3) induced upon DEX treatment combined with or without CHX when fusions with GR were used. White boxes: the respective promoter/transcript level was not significantly affected in the experiment, and this was documented in the respective study. Grey boxes: not tested/no results presented in the respective study. Note the differential regulation based on complex composition, e.g., for AtTTG1-GL3/EGL3/TT8-TT2/MYB5 complexes in [115]. AtTTG1-GL3/EGL3/TT8-PAP are known to be relevant in vegetative tissue (e.g., [115,118]). The following studies were used for compiling this figure and are indicated in the figure as numbers below the MBW factors: 1: [42], 2: [47], 3: [115], 4: [27], 5: [116], 6: [46], 7: [118], 8: [117]. *: wt vs. mutant or overexpressor (*ttg1*, *tt2*, *myb5*, *gl3egl3*, *gl3egl3tt8*, *gl3egl3tt8myc1*, MYBRNAi, *pap1*/pap1-D, Pro35S:*MYB113*). Black boxes mean in this case: The respective promoters are significantly downregulated in the respective mutants. #: upregulation effect of transcription upon overexpression of TTG1-GR or GL3-GR encoding constructs in *ttg1* or in the *gl3egl3* double mutant. ##: effect is also seen for GL3-GR when EGL3 is present in *gl3* single mutants (height sucrose concentration (3%) in this experiment); *F3H* and CHX were not tested in this experiment. x: fusion to GR in the presence of DEX and CHX leads to upregulation of the respective promoter tested (all in respective mutant background). *Pp*: *Physcomitrella patens* protoplasts *At*: *A. thaliana* (A7) protoplasts, siliques: young siliques, endoth.: endothelium, seedling +: seedlings treated with cycloheximide (CHX) when testing for the effect of overexpressing, e.g., AtTTG1-GR (glucocorticoid receptor) or GL3-GR, respectively. For chalaza and endothelium, the results are from the visual impression from GUS-stained seeds. Thick boxes around enzymes: LBGs. See, e.g., Figure 1 for the other abbreviations.

Progress made in the last decade in differentiating target-specific activity of MBW(TTG1) complexes is diverse and uses different experimental systems (see previous reviews for a developmental and hormonal embedding [20,113,114]). In Figure 4, we combine exemplary results from at set of studies. These analyze the regulation of the flavonoid biosynthesis’ core enzymes through differential sets of MBW(AtTTG1) components. The compilation highlights the need for systematic tissue-specific studies in this area of research. We apologize for all other examples not chosen here. 

Many studies have focused on the regulation of *DFR* through MBW(AtTTG1) complexes. This is not surprising as no *DFR* is expressed in absence of AtTTG1 as shown in *ttg1* mutants [14]. This leads to a complete blockage of the downstream pathway in these mutants [22]. On the level of bHLH factors, redundancy has been observed for AtTTG1-dependent traits. In this line, Zhang and co-workers observed a significantly reduced *DFR* expression as compared to the wildtype when analyzing seedlings. They also found a decrease of the *DFR* transcript level *gl3egl3* double mutants and an even lower level in *gl3egl3tt8* triple mutants (Figure 4, Study 1 [42]). 

Further downstream, the pathway, *BAN* expression was modulated in young siliques that carried constructs overexpressing AtTTG1, TT8 and TT2, which were fused to the glucocorticoid receptor (GR). These move into the nucleus upon dexamethasone (DEX) treatment. The increased *BAN* expression in these AtTTG1-GR and TT2-GR lines could be even more elevated when using cycloheximide (CHX) to block protein translation and thereby block secondary transcriptional effects possibly evoked by transcriptional activity of direct targets of AtTTG1 and TT2. This result was in contrast to TT8-GR induced *BAN* expression upon DEX treatment counteracted by CHX treatment suggesting a more complex regulation through TT8 (Figure 4, Study 2 [47]). In the same study, a strong induction by adding AtTTG1 to TT2-GL3/EGL3/TT8 dimer combinations was found. Here, *A. thaliana* protoplasts were used as the test system. Interestingly, TT2-EGL3 and TT2-TT8 did not require AtTTG1 for a significant induction of *BAN* transcription. MBW complexes with MYC1 could not activate the *BAN* promoter:GUS construct.

In one of the more comprehensive studies, mutants, promoter:GUS lines and GFP reporter constructs in the heterologous system *Physcomitrella patens* (protoplasts) were combined (Figure 4, Study 3 [115]). Most bHLH-PAP1/PAP2/TT2 dimers tested could activate *DFR*, *LDOX* or *BAN* in the absence of co-overexpressed AtTTG1 in *P. patens* or *A. thaliana* protoplasts with exceptions for TT2-MYC1 (*DFR*, *BAN*), TT2-GL3 (*BAN*) and TT2-TT8 for *LDOX* (Figure 4, Studies 2, 5–8 [46,47,116,117,118]). The selected studies cover TT2 dimers for *DFR* and *BAN*, PAP1 dimers for *DFR* and *LDOX* and PAP2 dimers for *DFR*. Endogenous proteins and low levels of AtTTG1 in the used protoplast might influence the results. Therefore, promoter binding studies will complement these results. First evidence of MYB-binding motives from Y1H studies is already available [33].

Xu and co-workers also acquired the expression of flavonoid biosynthetic genes in mutant background and compared it to the respective wildtype [115]. Not only the LBGs *DFR*, *LDOX* and *BAN* are downregulated in *tt2*, *tt8* and *ttg1* mutants, but also the EBGs *F3H* and *F3’H* in *tt2* and *ttg1* mutants. As MBW(AtTTG1) complexes affect transcriptional cascades, it is relevant to point out that direct activation of *DFR*, *LDOX* and again *BAN* is shown for AtTTG1-GR in siliques using the same DEX/CHX approach as described above. The regulation of F3’H is indirect for AtTTG1, but direct for GL3 (Figure 4, Study 4 [27]).

An important step towards understanding MBW(AtTTG1)-regulated flavonoid compositions will be tissue-specific studies using, e.g., promoter:GUS constructs for the pathways’ core enzymes as done for seeds [115]. Other important lines of experiments will be to bypass redundancies through systematic double and higher order mutants (e.g., [27,42]) and to analyze direct promoter binding.

## 7. MBW(AtTTG1) Complexes, Multilayered Regulatory Mechanisms at Work: A Possible Scenario

*Atttg1* mutant analysis suggests that *AtTTG1* is indispensable to regulate all *AtTTG1*-dependent traits in concert. It is assumed that AtTTG1, MYB and bHLH factors can form a ternary complex that acts as a transcriptional activator [31,35,38,119,120]. Interestingly, overexpression of the maize *R* gene in *Atttg1* mutant background could rescue all *AtTTG1*-dependent traits [121]. It has also been shown that bHLH-R2R3-MYB-dimers can activate tested promoters [122]. This might also have been the ancient status, as, e.g., mosses possess type IIIf bHLH factors, but no functional *TTG1* ortholog has been described to our knowledge [29,31]. GLABRA1 (GL1) and GL3 were shown to be able to activate CPC expression in *Arabidopsis* suspension cultures [122], and TT2 and TT8 overexpression is sufficient for *BAN* and LDOX activation, as well as PAP1 with EGL3 or TT8 for *LDOX* activation in A7 protoplasts, which express low levels of TTG1 according to the authors [47,117,118]. Therefore, the test system might not have been devoid of TTG1. Is this amount of putatively translated TTG1 sufficient to mediate a minimal required TTG1 function when the other factors are overexpressed? Here, studies in *ttg1* mutant background are required. Since bHLH and an R2R3-MYB factor can activate transcription without TTG1, what is the function of TTG1? This has not been solved yet, and many aspects require further investigation. Other possible scenarios are described below (see Section 8).

**Figure 5:**
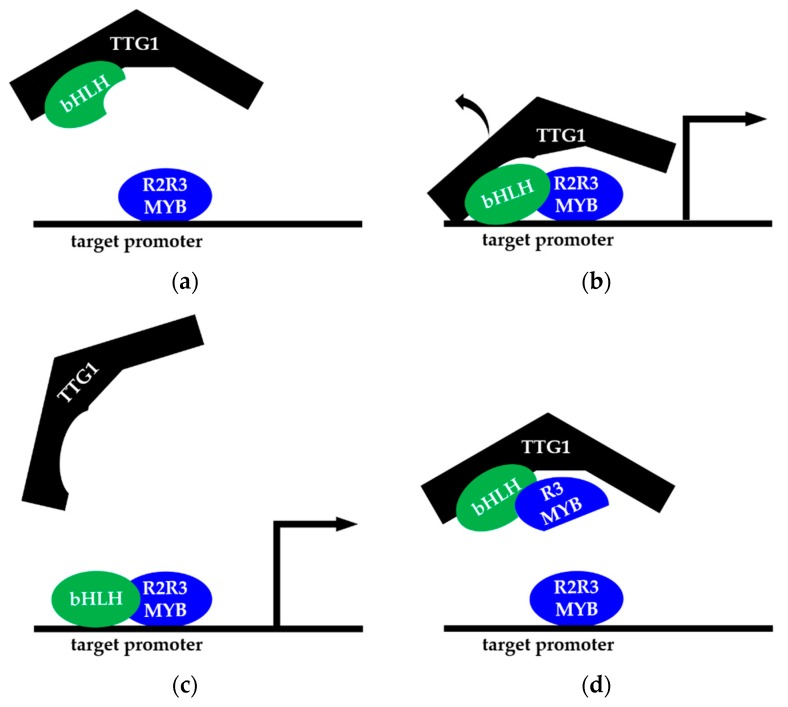
One speculative scenario illustrating the diverse missing links between MBW(AtTTG1) regulatory mechanisms. See the text for the details.

One possible function of AtTTG1 might be to shield the bHLH and R2R3-MYB factor from (negative) regulators. AtTTG1 was shown to be localized at promoters (e.g., *TRANSPARENT TESTA GLABRA2* (*TTG2*) and *CAPRICE* (*CPC*)) by semi-quantitative PCR of ChIP experiments [123]. It might modulate the chromatin at the target promoter to set the stage for the other factors (early in development). It might also be directed to target promoters by non-MYB, non-bHLH interaction partners (e.g., TTG2 [51]) or the following scenario might (Figure 5) occur:

Instead of ternary complex formation and binding to the promoter as a complex, a R2R3-MYB factor binds to a specific promoter. It cannot activate the promoter on its own, at least this was shown for the At*DFR* promoter and R2R2-MYB proteins of MBW(AtTTG1) complexes [46]. bHLH factors could dock to the WD40-platform TTG1 (Figure 5a). When the appropriate bHLH-TTG1 pair is available, it binds the R2R3-MYB factor, facilitated maybe by a bHLH binding motif at an appropriate distance, and the ternary complex is (at least transiently) formed (Figure 5b). Prior DNA binding of the R2R3-MYB factor might not be required. TTG1 possibly even dissociates again (Figure 5c), but was required for joining the bHLH-R2R3-MYB pair. This scenario also fits with the recently described mechanism of competition of TTG1 and GL1 for binding to GL3 [122].

The requirement of two transcription factors for target gene expression allows for integration of two or more signaling pathways through the regulation of protein abundance for both, the respective bHLH and R2R3-MYB factor. A third layer is added through the required presence of TTG1, which is needed in the scenario described above for initial complex formation. TTG1 might also be necessary to protect the bHLH factor from degradation or inactivation to achieve a threshold of molecules which jointly activate (with the respective MYB factor) the target gene′s transcription. Therefore, the rescue of *ttg1* mutants overexpressing the R gene from maize could be explained if R is not selective towards its *A. thaliana* R2R3-MYB interaction partner. Moreover, its protein levels, due to the overexpression, are high enough to compete with the negative regulation of the non-endogenous R protein.

In the above-described scenario, the competitors of the R2R3-MYB proteins for bHLH binding, R3-MYB factors (e.g., [56]) that do not act as transcriptional activators, could act very efficiently: they might bind to the TTG1-bHLH dimer also prior to DNA-binding (Figure 5d), evoke TTG1 release and thereby subject the bHLH protein to its negative regulators, e.g., the ubiquitin ligase UBIQUITIN-PROTEIN LIGASE 3 (UPL3)/KAKTUS [124]. The transcription of the target gene through an MBW(AtTTG1) complex is thereby counteracted.

The possible scenarios are still diverse and several might occur in different conditions, tissues and developmental stages. Many regulatory aspects are still to be uncovered for these well-investigated MBW(AtTTG1)-complexes. Careful hypothesis testing is required in the future and testing which mechanisms also apply for the other *AtTTG1*-dependent traits. Therefore, it is worth summarizing the regulatory mechanism stemming not only from work on the flavonoid-related *AtTTG1*-dependent traits. Which of these also act in flavonoid biosynthesis? Which evolved independently?

## 8. AtTTG1 and MBW(AtTTG1) Complexes: Modes of Regulation

Accumulating findings suggest that the AtTTG1-dependent regulation is more diverse than initially expected and point to a multilayered regulation of the flavonoid biosynthesis core enzymes. The relevance of TTG1 as a regulatory hub is pointed out by a plethora of regulatory pathways and mechanisms regulating upstream, downstream TTG1 and even within MBW(AtTTG1) complexes. This diverse AtTTG1-dependent trait regulation seems to require up- and down-stream partners either within MBW(AtTTG1) complexes or additional partners. As transcriptional regulators like AtTTG1 are not expected to expand with organism complexity to the extent as transcription factor families like R2R3-MYB or bHLH factors do [28], regulatory mechanisms centered on TTG1 might be conserved between TTG1-dependent traits. 

In the following, we are listing various modes of MBW(AtTTG1) regulation and provide example publications. On the level of bHLH/MYB factors, the relevance of post-translational modifications has been reviewed recently [125]. Many findings have extended the picture presented more than a decade ago, e.g., by Broun in 2005 [38], already suggesting some scenarios for which supportive results were found. Here, we do not differentiate between traits, as some of the mechanisms can be expected to be utilized for more than one trait, but this remains to be investigated in the future:

- Differential complex formation (for an example of differential target regulation in seeds: [115]).
- Insights from trichome and root hair patterning: activator/inhibitor and lateral inhibition, feedback loops’ de novo pattern formation (including cell-to-cell movement) [36,60,126,127].
- Trapping and deletion model [48,128].
- Competitors (R3-MYBs) [54,55,56,129,130].
- Competition of TTG1 and an R2R3-MYB factor for binding to a bHLH factor [122].
- Comparison of the ternary complex and bHLH-R2R3-MYBs activating target promoters [122].
- Phosphorylation, e.g., of TTG1 through BRASSINOSTEROID-INSENSITIVE 2 (BIN2) [49].
- Spatiotemporal accumulation and regulation of MBW(AtTTG1) components themselves (e.g., reviewed for TT8 in [119]) [131].
- Regulatory sequences in introns [112].
- Not only AtGL3 is shown to bind directly to regulated promoters, but also AtTTG1 can bind to the TTG2 promoter [123].
- Spatio-temporal expression of core enzymes and spatio-temporal accumulation of core substances throughout the developmental stages and in different tissues [11,20,131,132].
- Protein stability and possible proteasomal-dependent degradation of MBW(AtTTG1) components: the proteasomal-dependent degradation of TTG1 [128] and other MBW(AtTTG1) components have been shown [124,133,134]. In particular, PRODUCTION OF ANTHOCYANIN PIGMENT (PAP)2 and PAP1 are potential targets of COP1/SPA complexes that target transcription factors for proteasomal degradation [134]. Therefore, protein stability in dependence of light might have a regulatory influence on MBW(AtTTG1) complexes within flavonoid biosynthesis regulation [134]. UPL3 has been identified to mediate the proteasomal degradation of GL3 and ENHANCER OF GLABRA3 (EGL3) [124].
- Interaction with other proteins that are putative up- or down-stream modulators of MBW(AtTTG1) complex activity: e.g., interaction of TRANSPARENT TESTA1 (TT1) with TRANSPARENT TESTA2 (TT2) and PAP1, TEOSINTE BRANCHED 1, CYCLOIDEA, PCF1 (TCP)3 with R2R3-MYB factors and TTG2, SPL4, SPL5, BIN2, GEM or AT3G03960 with TTG1 [49,50,51,52,53,135,136,137]. Apart from post-translational modifications, interactors might interfere with MBW(AtTTG1) complex formation, lead to alternative complex formation and affect the MBW(AtTTG1) transcriptional activity as suggested for the TTG1-SPL4/5 and TTG2 interactions [51,52].
- Multilayered upstream (environmental) regulation, e.g., in the frame of sucrose and hormone signaling pathways [113].

To transfer this wealth of knowledge not only from trait to trait, but also between species, an evolutionary embedding of MBW(AtTTG1) components (here, emphasis on AtTTG1) is required.

## 9. *AtTTG1*: Evolutionary Embedding and Functional Orthologs 

**Figure 6:**
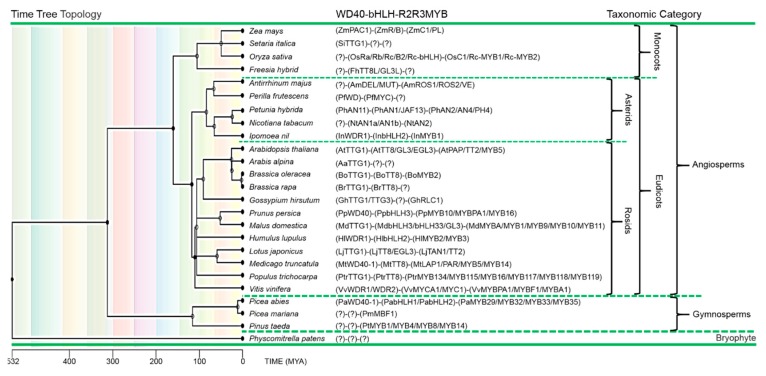
MBW proteins are common and conserved modules involved in regulating flavonoid biosynthesis throughout the plant kingdom. The phylogeny of selected land plants is reflected by time tree topology on the left (TimeTree (left) generated using the TimeTree database (www.timetree.org) [159]). Functionally-characterized MBW proteins from different plant species are listed in the middle (flavonoid pathway exclusive). Question marks indicate unidentified components. For a full list of proteins and references, please refer to Table 2.

The flavonoid biosynthetic pathway is found in a wide range of land plants, even in the bryophytes (mosses), and it has been suggested that synthesis of flavones, flavanones and flavonols may have evolved, first, to provide chemical messengers and then UV sunscreens [10,23]. Maize (*Zea mays*), petunia (*Petunia hybrida)* and snapdragon *(Antirrhinum majus)* emerged as the major models for the study of flavonoid biosynthesis, and genes encoding R2R3-MYB and bHLH proteins were identified as regulators of flavonoid structural genes, demonstrating broad conservation of this regulatory mechanism in these plants [121,138,139,140,141,142,143,144,145,146,147,148]. By then, the relationship between WDR proteins and the R2R3-MYB/bHLH transcriptional regulators had not been revealed. The first anthocyanin regulatory locus that was cloned from petunia, *AN11*, encodes a protein containing five WD40 repeats [149]. Further support for the formation of an MBW complex in plants came from interaction studies conducted in *A. thaliana* [40]. Similarly, the maize WDR PAC1 is specifically involved in the flavonoid pathway, but can complement all *ttg1* phenotypes in *A. thaliana*, and it became the first identified TTG1-like protein in monocots [138,150]. Later, WDR proteins were found to regulate the flavonoid biosynthetic pathway in other plants such as *Arabis alpina*, *Perilla frutescens*, *Ipomoea nil*, *Medicago truncatula*, *Malus domestica* and many others (see Figure 6 and Table 2) [138,151,152,153,154,155]. 

Regularly, new orthologs are identified, which might be of relevance for breeding purposes. Not only are allelism tests and rescue experiments within the respective species conducted (if applicable) to explore the ortholog’s function, when mutants or *TTG1* variants are identified. More often, the function of the orthologs is estimated by using the model species *A. thaliana*. In cross-species rescue experiments, *TTG1* orthologs from other species are expected to take over *AtTTG1* function at least in part within respective MBW(TTG1) complexes. This year, for example, ectopic expression of *SiTTG1* (a newly identified *AtTTG1* ortholog in *Setaria italica*) in the *A. thaliana* *ttg1-13* background was shown to fully rescue the glabrous trichome and the flavonoid phenotype. This suggests that *SiTTG1* is a member of flavonoid biosynthesis regulators in monocots [156]. Another example is *BrTTG1*, which was isolated from a brown-seeded hairy *Brassica rapa* and found to functionally complement an *A. thaliana ttg1* mutant, while another ortholog, isolated from *Brassica rapa* yellow-seeded glabrous germplasm, was not functional [157]. 

It needs to be mentioned that promoter sequences and MBW components might have differentially evolved in the respective other species and thereby led to shifts in MBW(TTG1) function. Nevertheless, newly-identified orthologs also provide novel insights into the evolutionary aspects of MBW(TTG1)s.

MBW complexes have been identified as common and conserved flavonoid biosynthesis regulators, similarly reported for various land plants; although in several cases, not all components have been identified or characterized (Figure 6 and Table 2). Recently, MBW complexes (PaWD40-1-PabHLH1/2-PaMYB29/32/33/35) were characterized in Norway spruce (*Picea abies*) and shown to be involved in the regulation of the flavonoid biosynthesis pathway [94]. This reveals a full MBW regulator in gymnosperms, which were previously thought to be devoid of *TTG1* orthologs [31]. 

MBW complexes determine the spatiotemporal expression of flavonoid biosynthesis target genes that account for tissue-specific accumulation of flavonoids. Some MBW complexes from monocots can control the expression of enzymes of the entire pathway, while others specifically control late flavonoid biosynthesis genes in eudicots [158]. Nevertheless, the bHLH interaction motif ([DE]Lx_2_[RK]x_3_Lx_6_Lx_3_R) found in R2R3-MYB members of MBW complexes is highly conserved among higher plant species [125], suggesting that at least MYB and bHLH interactions arose early during the event of land plant evolution.

Interestingly, in the rosid clade, besides regulation of the flavonoid biosynthesis pathway, combinatorial MBW complexes evolved several extra functions: for instance, trichome patterning, root hair patterning and seed coat mucilage production in *A. thaliana* [41,42,43,45,128,160,161,162,163,164,165] or *Arabis alpina* [155] and seed hair formation in cotton (*Gossypium hirsutum*) [166,167,168,169]. These observations imply that a common regulatory MBW module has been adapted for controlling specific epidermal cell fates in rosids.

However, such pleiotropic functions of MBW complexes have neither been observed in the asterid clade nor in monocots. Based on this, a speculation is raised: new roles of MBW complexes in controlling epidermal cell fate may have diverged since the evolutionary separation of these major plant groups, although the details of this are still not clear [170,171]. This is supported by the findings that multicellular trichome and conical cell formation in asterids, like *Antirrhinum* and *Solanaceae* species, are regulated by MIXTA-like R2R3-MYB-related proteins in which the bHLH interaction motif ([DE]Lx_2_[RK]x_3_Lx_6_Lx_3_R) is devoid. *MIXTA* gene overexpression in rosids does not affect trichome formation [171,172,173]. AtGL1, a trichome patterning specific-R2R3-MYB protein, was grouped phylogenetically together with AtPAP and AtTT2 that act in the regulation of the flavonoid biosynthesis pathway. This clade is distinct from the MIXTA-like regulators branch [72,174,175]. It is assumed that duplication and subsequent divergence, as known for other protein families [28], has been the driving force to evolve new roles of MBW complexes and other epidermal cell fates in rosids as compared to asterids [174].

Such divergence might have been revealed in *Beta vulgaris* where the dominating pigment is betalain [176]. Betalain accumulation was shown to be regulated through *BvMYB1* (gene at the beet *Y* locus), an R2R3-MYB factor. The authors speculate that this can be seen as an evolutionary event allowing betalains to functionally replace anthocyanidins [177]. Molecular evolution seems to have occurred. Similar to the AmMIXTA R2R3-MYB-related protein, BvMYB1 cannot interact with bHLH proteins. When mutating the interaction motif to the consensus plus an additionally conserved amino acid, bHLH interaction was reconstituted [177]. With the genomic sequence, RNAseq data and functional categorization at hand, a deeper insight into the changes and evolution of protein binding, as well as *cis* regulatory motives is to be expected in the future [178].

## 10. Perspective

“Who does what, where, when and why?” There is constant progress in understanding the spatiotemporal regulation mediated by MBW(TTG1) complexes in the model species *A. thaliana*, but also in fruits and crops. Understanding changing flavonoid compositions, e.g., for the benefit of pre- and post-harvest fruit quality in dependence of developmental and environmental cues (see, e.g., [225,226]) includes understanding the activity of major regulators of the flavonoid biosynthesis pathway like the MBW(TTG1) complexes. However, also biotechnological engineering of the flavonoid biosynthesis might benefit from recent findings on the pathways’ regulation [116]. 

Why plants accumulate specific flavonoid compositions, especially a wide range of specific derivatives in different tissues throughout development, is still not fully understood, and the function of individual substances, as well as mixtures could be a central question driving future research.

## 11. Methods

Seven hundred and sixty reviews containing “flavonoid(s)” in the title were extracted with EndNote X8 (Clarivate Analytics, Philadelphia, PA, USA) from the PubMed and Web of Science databases in October 2017 and sorted by topic. The same databases were searched with EndNote X8 for an initial overview of literature related to TTG1/URM23 (search for full gene name and abbreviation in title/abstract/keywords). Additional references were manually selected. We apologize to any researcher who′s work on the topic this review could not cover.

## Figures and Tables

**Table 1:** Comprehensive list of *A. thaliana ttg1* mutants with documentation on seed pigmentation.

Allele	Background	Mutagenesis	Mutation	Phenotype	References
*ttg1-1*	L*er*	EMS	Q317stop	*tt*, *g*	[15,34]
*ttg1-9*	Col-0	EMS	S282F	*tt*, *g*	[34,95,96]
*ttg1-10*	Ws	EMS	g->a (5′UTR)	*tt*, (*g*)	[97]
*ttg1-11*	Col-0	EMS	G149R	*tt*, *g*	[97]
*ttg1-12*	Col-0	EMS	G43R	*tt*, *g*	[97]
*ttg1-13*	RLD1	fast neutrons	deletion	*tt*, *g*	[97]
*ttg1-15*	Antwerp-1	Kranz collect.	S310stop	*tt*, *g*	[34] *
*ttg1-16*	Enkheim-1	Kranz collect.	S310stop	*tt*, *g*	[34] ^1^
*ttg1-17*	Enkheim-1	Kranz collect.	S310stop	*tt*, *g*	[34] ^1^
*ttg1-18*	Enkheim-1	Kranz collect.	S310stop	*tt*, *g*	[34] ^1^
*ttg1-19*	Enkheim-1	Kranz collect.	W183stop	*tt*, *g*	[34] ^1^
*ttg1-20*	Enkheim-1	Kranz collect.	S30C, S310stop	*tt*, *g*	[34] ^1^
*ttg1-21*	Col-0	T-DNA ^2^	exon 1	*tt*, NA	[11,98]
*ttg1-21-CI* *	Col	carbon ion irradiation	exon 1 (CI)	*tt*, NA	[99]
*ttg1-22*	Col-0	T-DNA ^2^	exon 1	*tt*, NA	[11,98]
*ttg1-213*	NA	NA	W183stop	*tt*, *g*	[100]
*urm23*	*gl1-2* (Col-1)	EMS	G302E	((*g*))	[100]
*ttg1* (Est)	Est-1	EMS	S101F	*tt*, *g*	[101]
*ttg1-P313*	Col-4	T-DNA ^3^	insertion (ND)	*g*	[102,103]
*ttg1-P416*	Col-4	T-DNA ^3^	insertion (Chr5: 8371365..8371665)	*tt*, *g*	[102,103]
*ttg1-SK31268*	Col-4	T-DNA ^3^	insertion (ND)	*tt*, *g*	[102,103]
*ttg1-SK41546*	Col-4	T-DNA ^3^	insertion (Chr5: 8371188..8371488)	*tt*, *g* ^#^	[102,103]

* Note that *ttg1-21* has been assigned twice and “-CI” was added here for clarification, CI = carbon ion-induced chromosomal rearrangement (breakage in exon 1 of TTG1 on chromosome 5 and rejoining with chromosome 3), *tt*: *transparent testa* phenotype, *g*: *glabra* phenotype, severity of glabrous phenotype: *g* > (*g*) > ((*g*)); ^1^ origin: Kranz collect. = Kranz collection; ^2^ GABI (“Genomanalyse im biologischen System Pflanze”) -Kat (“Kölner Arabidopsis T-DNA”) lines [98]; ^3^ SK (Saskatoon) population [103]: activation tagging lines (insertion of pSKI015 [104]); ^#^ enlarged trichome cells usually with no aerial extension, but sometimes stubby un-branched trichomes. Please note that not in all cases has the function been linked to the mutation. Not all studies conducted a rescue experiment or tested for allelism with a previously established mutant. Please refer to the references for more details. Col: Columbia, L*er*: Landsberg *erecta*, Est: Estland, EMS: Ethyl methanesulfonate (used for mutagenesis), T-DNA: transfer-DNA, UTR: untranslated region, NA: no information available, *urm*: *unarmed*, *gl1*: *glabra1*, ND: position of insertion was not determined in the respective study.

**Table 2:** MBW (R2R3-MYB-bHLH-WDR) proteins (putatively) regulating flavonoid biosynthesis from different plant species.

Species	MBW Proteins			References
	WDR	bHLH	R2R3-MYB	
*Zea mays*	ZmPAC1	ZmR/B ZmIN1 (repressor)	ZmC1; ZmPL ZmP1 (independence of WDR and bHLH)	[58,82,83,138,144,145,179,180,181]
*Setaria italica*	SiTTG1			[156]
*Oryza sativa*		OsRa/Rb/Rc OsB2 OsRc-bHLH	OsC1; OsRc-MYB1/2	[29,182,183,184]
*Freesia hybrida*		FhTT8L FhGL3L		[185]
*Antirrhinum majus*		AmDEL AmMUT	AmROS1/2; AmVE AmMYB308/330 (repressors)	[147,186,187,188]
*Perilla frutescens*	PfWD (cytosol)	PfMYC		[151]
*Petunia hybrida*	PhAN11 (cytosol)	PhAN1 PhJAF13	PhAN2/4; PhPH4 PhMYB27 (repressor)	[139,140,141,142,143,147,149,175]
*Nicotiana tabacum*		NtAN1a/b	NtAN2	[189]
*Ipomoea nil*	InWDR1 InWDR2 *	InbHLH2 InbHLH1/3 *	InMYB1 InMYB2/3 *	[152]
*Arabidopsis thaliana*	AtTTG1	AtTT8 AtGL3 AtEGL3 AtMYC1 *	AtPAP1/2; AtMYB113/114; AtTT2; AtMYB5 AtMYB4 (repressor)	[15,27,34,42,46,47,165,190,191,192,193,194,195,196]
*Arabis alpina*	AaTTG1			[155]
*Brassica oleracea*	BoTTG1	BoTT8 BoEGL3 *	BoMYB2 BoMYB12 * BoTT2 *	[197]
*Brassica rapa*	BrTTG1	BrTT8		[198,199]
*Gossypium hirsutum*	GhTTG1/3	GhDEL61/65 * GhMYC1 *	GhRLC1	[166,167,168,169]
*Prunus persica*	PpWD40	PpbHLH3	PpMYB10.1/10.3; PpMYBPA1; PpMYB16 PpMYB111 (a repressor)	[200,201,202]
*Malus domestica*	MdTTG1	MdbHLH3 MdbHLH33 MdGL3	MdMYB1/9/10/11; MdMYBA	[153,203,204]
*Humulus lupulus*	HlWDR1	HlbHLH2	HlMYB2/3 HlMYB7 (a repressor)	[205]
*Lotus japonicus*	LjTTG1	LjTT8, LjEGL3 LjRHL1 *	LjTAN1; LjTT2a/b/c	[206,207]
*Medicago truncatula*	MtWD40-1	MtTT8 MtEGL3 *	MtLAP1; MtPAR; MtMYB5/14 MtMYB2 (a repressor)	[154,208,209,210,211,212]
*Populus trichocarpa*	PtrTTG1	PtrTT8	PtrMYB134/115/116/117/118/119; PtrMYB182 (a repressor)	[213,214,215]
*Vitis vinifera*	VvWDR1/2	VvMYCA1 VvMYC1	VvMYBPA1; VvMYBF1; VvMYBA1	[216,217,218,219,220]
*Picea abies*	PaWD40-1	PabHLH1/2 PabHLH3 *	PaMYB29/32/33/35 PaMYB30/31/34 *	[94]
*Picea mariana*			PmMBF1	[221]
*Pinus taeda*			PtMYB1/4/8/14	[222,223]
*Physcomitrella patens*		PpRSL1/2 *(rhizoid development)		[29,224]

* Functions in flavonoid biosynthesis regulation remain to be confirmed. Note, not for all proteins have full MBW complexes been described (so far). Some might turn out not to be present in these species as MBW complexes.

## Abbreviations

Aa	*Arabis alpina*
Am	*Antirrhinum majus*
AN (e.g., AN11)	ANTHOCYANIN (e.g., ANTHOCYANIN11)
ANR	ANTHOCYANIDIN REDUCTASE
ANS	ANTHOCYANIDIN SYNTHASE
At, *A. thaliana*	*Arabidopsis thaliana*
B	BOOSTER
BAN	BANYLUS
bHLH	basic HELIX-LOOP-HELIX
BIN2	BRASSINOSTEROID-INSENSITIVE 2
Bo	*Brassica oleracea*
Br	*Brassica rapa*
C1	COLORLESS1
ChIP	Chromatin Immunoprecipitation
Col	Columbia
COP1	CONSTITUTIVELY PHOTOMORPHOGENIC 1
CPC	CAPRICE
DEL	DELILA
DFR	DIHYDROFLAVONOL 4-REDUCTASE
EBG	early biosynthetic gene
EGL3	ENHANCER OF GLABRA3
EMS	Ethyl methanesulfonate
Est	Estland
ETC	ENHANCER OF TRY AND CPC1
Fh	*Freesia hybrida*
GEM	GL2-EXPRESSION MODULATOR
*g*	*glabra*
Gh	*Gossypium hirsutum*
GL1	GLABRA1
GL3	GLABRA3
Hl	*Humulus lupulus*
In	*Ipomoea nil*
IN1	INTENSIFIER1
JAF	JOHNANDFRANCESCA
LAP1	*LEGUME ANTHOCYANIN PRODUCTION 1*
LBG	late biosynthetic gene
LDOX	LEUCOANTHOCYANIDIN DIOXYGENASE
L*er*	Landsberg *erecta*
Lj	*Lotus japonicus*
Md	*Malus domestica*
Mt	*Medicago truncatula*
MUT	MUTABILIS
MYB	MYELOBLASTOSIS (homolog)
MYBL	MYB-like
MYBPA	MYB transcription factors regulating PA synthesis
MYC	MYELOCYTOMATOSIS (homolog)
Nt	*Nicotiana tabacum*
Os	*Oryza sativa*
P1	PERICARP COLOR1
Pa	*Picea abies*
PAC1	PALE ALEURONE COLOR1
PAP	PRODUCTION OF ANTHOCYANIN PIGMENT
PAR	PROANTHOCYANIDIN REGULATOR
PAs	proanthocyanidins
Pf	*Perilla frutescens*
Ph	*Petunia hybrida*
PH	pH
PL	PURPLE LEAF
Pm	*Picea mariana*
Pp	*Physcomitrella patens*
Pp	*Prunus persica*
Pt	*Pinus taeda*
Ptr	*Populus trichocarpa*
R	RED
RHL1	ROOT HAIRLESS1
RLC	RED LEAF COTTON
ROS	ROSEA
RSL	ROOT HAIRT DEFFECTIVE SIX-LIKE
Si	*Setaria italica*
SPA	SUPPRESSOR OF PHYA-105
SPL	SQUAMOSA PROMOTER BINDING PROTEIN-LIKE
TAP	TRANSCRIPTION-ASSOCIATED PROTEIN
TCP	TEOSINTE BRANCHED 1, CYCLOIDEA, PCF1
TCL	TRICHOMELESS
T-DNA	transfer-DNA
TRY	TRIPTYCHON
*tt*	*transparent testa*
TT1	TRANSPARENT TESTA1
TT2	TRANSPARENT TESTA2
TT8	TRANSPARENT TESTA8
TTG1	TRANSPARENT TESTA GLABRA 1
TTG2	TRANSPARENT TESTA GLABRA 2
UPL3	UBIQUITIN-PROTEIN LIGASE 3
URM23	UNARMED23
UTR	untranslated region
VE	VENOSA
Vv	*Vitis vinifera*
WDR	WD40 repeat
WER	WEREWOLF
Zm	*Zea mays*
